# SDF4 Is a Prognostic Factor for 28-Days Mortality in Patients With Sepsis *via* Negatively Regulating ER Stress

**DOI:** 10.3389/fimmu.2021.659193

**Published:** 2021-07-13

**Authors:** Tingting Zhu, Qun Su, Cuili Wang, Lingling Shen, Hongjun Chen, Shi Feng, Xiaofeng Peng, Siyu Chen, Yucheng Wang, Hong Jiang, Jianghua Chen

**Affiliations:** ^1^ Kidney Disease Center, The First Affiliated Hospital, College of Medicine, Zhejiang University, Hangzhou, China; ^2^ Key Laboratory of Nephropathy, Hangzhou, China; ^3^ Institute of Nephropathy, Zhejiang University, Hangzhou, China; ^4^ Critical Care Medicine Department, The First Affiliated Hospital, College of Medicine, Zhejiang University, Hangzhou, China

**Keywords:** sepsis, prognosis, SDF4, gene co-expression network, endoplasmic reticulum stress, CLP

## Abstract

Sepsis is a heterogeneous syndrome induced by infection and results in high mortality. Even though more than 100 biomarkers for sepsis prognosis were evaluated, prediction of patient outcomes in sepsis continues to be driven by clinical signs because of unsatisfactory specificity and sensitivity of these biomarkers. This study aimed to elucidate the key candidate genes involved in sepsis response and explore their downstream effects based on weighted gene co-expression network analysis (WGCNA). The dataset GSE63042 with sepsis outcome information was obtained from the Gene Expression Omnibus (GEO) database and then consensus WGCNA was conducted. We identified the hub gene *SDF4* (stromal cell derived factor 4) from the M6 module, which was significantly associated with mortality. Subsequently, two datasets (GSE54514 and E-MTAB-4421) and cohort validation (n=89) were performed. Logistic regression analysis was used to build a prediction model and the combined score resulting in a satisfactory prognosis value (area under the ROC curve=0.908). The model was subsequently tested by another sepsis cohort (n=70, ROC= 0.925). We next demonstrated that endoplasmic reticulum (ER) stress tended to be more severe in patients PBMCs with negative outcomes compared to those with positive outcomes and *SDF4* was related to this phenomenon. In addition, our results indicated that adenovirus-mediated *Sdf4* overexpression attenuated ER stress in cecal ligation and puncture (CLP) mice lung. In summary, our study indicates that incorporation of *SDF4* can improve clinical parameters predictive value for the prognosis of sepsis, and decreased expression levels of *SDF4* contributes to excessive ER stress, which is associated with worsened outcomes, whereas overexpression of *SDF4* attenuated such activation.

## Background

Sepsis, a syndrome of physiologic, pathologic, and biochemical abnormalities induced by infection, is a major cause of mortality among critical ICU patients (1). The annual incidence rate of sepsis ranges from 437 to 1031 per 100000 person-years, while its hospital mortality rate remains at a high level of over 25% even with modern surveillance and monitoring, prompt initiation of therapy, and advances in the support of failing organs (2–5). In addition to rapid diagnosis, one of the clinical dilemmas that we faced is that once a sepsis diagnosis is made and appropriate treatment is taken, it is often difficult to distinguish between a positive and a negative prognosis. In the past thirty years, more than 100 markers have been evaluated for sepsis prognosis, including cytokine/chemokine biomarkers, cell and receptor biomarkers, coagulation biomarkers, vascular endothelial damage biomarkers, vasodilation biomarkers, organ dysfunction biomarkers, and acute-phase protein biomarkers (6, 7). Nevertheless, the prediction of patient outcomes in sepsis continues to be driven by clinical signs because of the unsatisfactory specificity and sensitivity of these currently available biomarkers. Therefore, it is vital to research novel biomarkers for a better prediction of sepsis progression to improve the prognosis of sepsis patients.

In recent years, analysis of differentially expressed genes (DEGs) detected by high-throughput sequencing, followed by enrichment analyses to established functional pathways were performed in several studies to improve prognostic accuracy and establish the underlying molecular processes (8–10). However, host responses during sepsis are highly heterogeneous, which involve multidimensional networks of molecules and cells. Therefore, the sensitivity of individual genes might be low while network analysis might be more informative, especially when the set of available expression data is large (11, 12). Weighted correlation network analysis (WGCNA) is a systems biology approach for describing the correlation patterns among genes across microarray samples (13). It is widely applied to various biological contexts, such as tumor (14–16), metabolic disease (17), neurological disorders (18), and autoimmune disease (19, 20). WGCNA has also been used to analyze correlation patterns in sepsis. These research efforts focused on inflammatory cells *in vitro* (21), long non-coding RNA (22), transcription factors and miRNA (23) based on samples with or without sepsis, but did not distinguish between patients with positive outcomes from those with negative outcomes. A recent study identified two transcription factors, CEBPB and ETV6, associated with mortality outcome *via* WGCNA (24).

In the present study, we performed a WGCNA analysis to identify co-expression modules relating to the outcome of sepsis. KEGG enrichment analysis was performed on the interested modules and a hub gene was identified. We validated the hub gene in ICU patients in our hospital and conducted a logistic regression analysis to build a prediction module. Downstream effects of the hub gene were then explored, which might provide potential targets for sepsis treatment in the future.

## Materials and Methods

### RNA Sequencing Data and Data Pre-Processing

The publicly available gene expression profile GSE63042 was downloaded from the Gene Expression Omnibus (GEO) database (https://www.ncbi.nlm.nih.gov/geo/). The dataset contained 129 peripheral blood RNA samples, including 23 with systemic inflammatory response syndrome (SIRS) and 106 with sepsis. Furthermore, the sepsis patients could be further categorized into non-survivors and survivors at 28 days. Details of samples preparation and RNA sequencing method were elaborated in the previous study (25). 28 sepsis non-survivors and 78 sepsis survivors were used for the follow-up WGCNA analysis after filtering low-expression gene (mean RPM of all samples less than 0.5) and removing duplicate genes randomly.

### Construction of the Gene Co-Expression Network and Module Detection *via* WGCNA

WGCNA was used to assess the weighted gene co-expression network and its module membership under the R platform (13). First, we selected the soft threshold (β) for network construction in the WGCNA algorithm. The soft threshold makes the adjacency matrix to be a continuous value between 0 and 1 so that the constructed network conforms to the power-law distribution and is closer to the real biological network state. Then, a topological overlap matrix (TOM) similarity function was used to convert the matrix to a TOM. Co-expression genes were assigned to modules *via* dynamic minimum tree-cutting arithmetic.

### Identification of Trait-Related Modules

Similar modules were merged into one and then 11 total modules were obtained. The module eigengenes (ME), which represented the expression level for each module, were calculated. Then, the correlation between ME and clinical traits in each module was calculated, and a *P*-value < 0.05 was considered to be statistically significant for determining trait-related modules. There were two parameters to further access the relationship between module and trait. Gene Significance (GS) represented the correlation between individual genes and traits and Module Membership (MM) calculated the association between individual genes and the MEs of the module.

### Differentially-Expressed Genes (DEGs) in Trait-Related Modules and the Identification of a Hub Gene

DEGs analysis between sepsis survivors and sepsis non-survivors was performed by authors of GSE63042. There are 1,238 genes differentially expressed (1,099 annotated) between sepsis survivors and sepsis non-survivors (25). Then, the M6 module, which included the majority of DEGs, was imported into Cytoscape with their weighted correlations. We identified hub gene with the following criteria:1) DEGs; 2) GS > 0.2, MM > 0.8; 3) highest 20 Maximal Clique Centrality(MCC)value calculated by the Cytohubba package in Cytoscape (26).

### KEGG Analysis

DAVID, an online bioinformatics tool, is designed to predict a large number of gene function (27). Kyoto Encyclopedia of Genes and Genomes (KEGG) pathways were used to represent the detailed information of the biological functions of genes in known signaling or metabolic pathways. Therefore, we used DAVID to visualize the gene enrichment of pathways (P < 0.05).

### Dataset Validation

In order to verify the expression of hub genes with larger sample size, two datasets (GSE54514 and E-MTAB-4421) (28, 29) with sepsis prognosis information were downloaded from GEO and ArrayExpress (https://www.ebi.ac.uk/arrayexpress/), respectively. In GSE54514, we only selected the samples collected within the initial 24 h of admission to the ICU to be consistent with the GSE63042 and E-MTAB-4421. The sepsis criteria were consistent in three datasets. Then, the expression of the hub gene was compared between sepsis survivors and non-survivors.

### Patients and Blood Sample Collection

Studies involving human participants were reviewed and approved by the Research Ethics Committee of the First Affiliated Hospital, College of Medicine, Zhejiang University. The patients/participants (or their next of kin) provided written informed consent to participate in this study. Patients over 18 years of age diagnosed with sepsis in the intensive care unit (ICU) were included in the study. These patients had been hospitalized at the First Affiliated Hospital, College of Medicine, Zhejiang University between 1 March 2020 and 30 May 2021. Sepsis was defined according to The Third International Consensus Definitions for Sepsis and Septic Shock (Sepsis-3) [1]. Detailed information of the selected patients was further reviewed by a clinician. Blood samples were obtained within the first 24 h following admission. Peripheral blood mononuclear cells (PBMCs) separation was performed within 3 h following collection. Patients status was monitored up to 28 days after the initial blood draw.

### RNA Extraction, cDNA Synthesis, and Real-time Quantitative PCR

PBMCs RNA was isolated *via* TRIzol^®^ reagent (Invitrogen, CA, USA). PrimeScript™ II Reverse Transcriptase (Takara, Shiga, Japan) was used to reverse transcribe 1000 ng of RNA to cDNA for each sample. Before qPCR, we diluted first-strand cDNA synthesis products 1:4 for each sample. A two-step PCR reaction was performed as follows: pre-denaturation, 95°C for 30 seconds; PCR reaction, denaturation at 95°C for 5 s, annealing at 60°C for 31 s for 40 cycles; dissociation at 95°C for 15 s, 60°C for 1 min, and 95°C for 15 s. The relative mRNA expression of target genes was normalized to GAPDH in the same sample. Results were expressed as fold change in expression, and values were calculated as the ratio of target gene expression to control gene expression. Gene-specific primers were designed *via* Primer-Blast (**Supplementary Table 1**). The efficiency of the primers was estimated by Ct< 30 and no multiple T_m_ peaks. Real time-PCR reactions were carried out using SYBR Green reagent and the CFX96™ Real-Time PCR Detection System (Bio-rad, CA, USA).

### Transmission Electron Microscopy

PBMCs were fixed in 2.5% (w/v) glutaraldehyde overnight, washed with PBS three times, stained with 1% osmium tetroxide, and then counterstained with 2% (w/v) uranyl acetate. After dehydration in a gradient series of ethanol (50% for 15min, 70% for 15min, 90% for 15min, and 100% for 20min) and 100% acetone for 20min, samples were embedded in Epon 812 (Electron Microscopy China, Beijing). Specimens were then cut into sections of 70 nm in thickness using the Leica EM UC7 microtome (Leica Biosystems) and ultrathin sections were visualized *via* transmission electron microscopy (FEI, Tecnai G2 Spirit Bio TWIN).

### Flow Cytometry

PBMCs were first fixed and permeabilized with IC Fixation Buffer (Invitrogen) and Permeabilization Buffer (Invitrogen). Cells were then incubated with specific primary antibodies against ATF6(Invitrogen, PA5-72554), CHOP (Invitrogen, PA5-35129), and GRP78 (Invitrogen,14-9768-82) for 1 h at 4°C. Isotype antibodies that lack specificity to the target but possess the class and type of the relevant primary antibodies served as a negative control. Cells were then washed with Permeabilization Buffer followed by incubation with APC labeled goat anti-rabbit IgG cross-adsorbed secondary antibody (Invitrogen, A-10931) and FITC labeled goat anti-mouse IgG cross-absorbed secondary antibody (Invitrogen, F-2761) for 1 h at 4°C in dark. After washing, cells were resuspended in Flow Cytometry Staining Buffer, and data were acquired by flow cytometry in a FACSCanto II device (BD Bioscience). Further analysis was performed in FlowJo.

### Cell Immunofluorescence

Freshly-isolated PBMCs were used for immunofluorescence assays. PBMCs were plated on poly-L-lysine-coated coverslips. 4% paraformaldehyde was used to fix the cells for 15 min. Cells were permeabilized with 0.3% TritonX-100 for 5 min and blocked with 3% BSA for 1 h at room temperature. Then, cells were incubated with primary antibody against ATF6 (1:200 dilution in 3% BSA), CHOP (1:100 dilution in 3% BSA), and GRP78 (1:200 dilution in 3% BSA) for 40 min. After three washes with PBS, cells were incubated with a 1:1000 dilution in 3% BSA of Alexa-488 or -594 conjugated secondary antibody. Stained cells were given a final wash and mounted with Prolong Gold antifade reagent with DAPI (Invitrogen). Images were taken with a 40× objective lens by immunofluorescence microscopy (Lacia, DM4000).

### Adenovirus Production and Adenovirus Delivery

The adenovirus used to overexpress Sdf4 in mice were purchased from Hanbio Biotechnology Co., Ltd. (Shanghai, China). Mice were intratracheally treated with 10^10^ plaque-forming units (PFU) of recombinant Sdf4 adenovirus (AdSdf4), EGFP adenovirus (AdCon) or PBS and allowed to recover for 72 hours before CLP.

### Cecal Ligation and Puncture (CLP) Model

All mice were maintained in the animal center of Zhejiang University according to animal care regulations. Research Ethics Committee of the First Affiliated Hospital, College of Medicine, Zhejiang University approved the experimental protocols. All experiments were carried out in accordance with the NIH Guide for the Care and Use of Laboratory Animals. Six to eight weeks old C57BL/6 wild type male mice were used for modeling. Polymicrobial sepsis was induced *via* CLP as previously described (30). Briefly, abdominal anesthesia was performed with 1% sodium pentobarbital (80mg/kg). Each abdomen was disinfected using 75% alcohol, and the skin was paralleled on the right side of the midline of the abdomen (operator’s field of vision). 75% cecum was ligated with 6/0 line and punctured using a 22G needle. 1–2mm3 of fecal material was expelled from the cecum into the peritoneal cavity, the cecum was returned to the abdominal cavity, and the abdominal muscle layer and skin were sutured layer by layer with a 4/0 line. The mice in the sham operation group were not subjected to cecal ligation and puncture, but all other procedures were the same as those for the experimental group. Mice were sacrificed 6 h,12 h, 18 h and 24h following CLP and lung was harvested.

### Western Blotting

Mice lungs were homogenized using RIPA containing a protease and phosphatase inhibitor (Roche). Protein content was quantified using a PierceTM BCA Protein Assay Kit (Thermo) and proteins were separated *via* SDS-PAGE with 10% acrylamide and transferred into PVDF membranes (Millipore, Burlington, MA, USA). Next, blots were blocked with 5% milk and probed with specific primary antibodies against SDF4 (1:500 dilution in 3% milk, Santa, sc-393930), ATF6, GRP78 and CHOP (1:1000 dilution in 3% milk) overnight. The membranes were then washed with TBST and incubated with horseradish peroxidase-conjugated secondary antibodies at room temperature for 1 h. Next, target proteins were detected using chemiluminescence *via* Bio-rad ChemiDoc MP and normalized to β-actin.

### Histology and Immunostaining

Formalin-fixed and paraffin-embedded lung tissues were cut into sections of 3 um in thickness for immunohistochemistry (IHC). 4% paraformaldehyde-fixed lung tissues were cut into sections of 6 um in thickness for immunofluorescence (IF) staining. IHC and IF were performed as previously (31). The primary antibody used were SDF4 (1:100 dilution in 3% BSA), ATF6 (1:200 dilution in 3% BSA), GRP78 (1:200 dilution in 3% BSA) and CHOP (1:200 dilution in 3% BSA). Images were acquired using Leica DM4000 microscope and Nikon A1 Ti confocal microscope.

### Statistical Analyses

The data are expressed as mean ± SD. Student’s *t*-tests and chi-square test were used for comparisons between two groups. A logistic regression model was conducted to create a combined predictive score. Diagnostic ability was evaluated using a receiver operating characteristic (ROC) curve and the area under the ROC curve (AUC) for individual and combined scores. The association between *SDF4* and *GRP78* or *ATF6* expression was examined by Pearson correlation analysis. Significance was set at *P* < 0.05. Statistical analyses and diagrams were performed using SPSS 20 (IBM) and GraphPad Prism 7.0 (GraphPad Software, CA, USA).

## Results

### Weighted Co-Expression Network Construction and Module Identification

**Figure 1:**
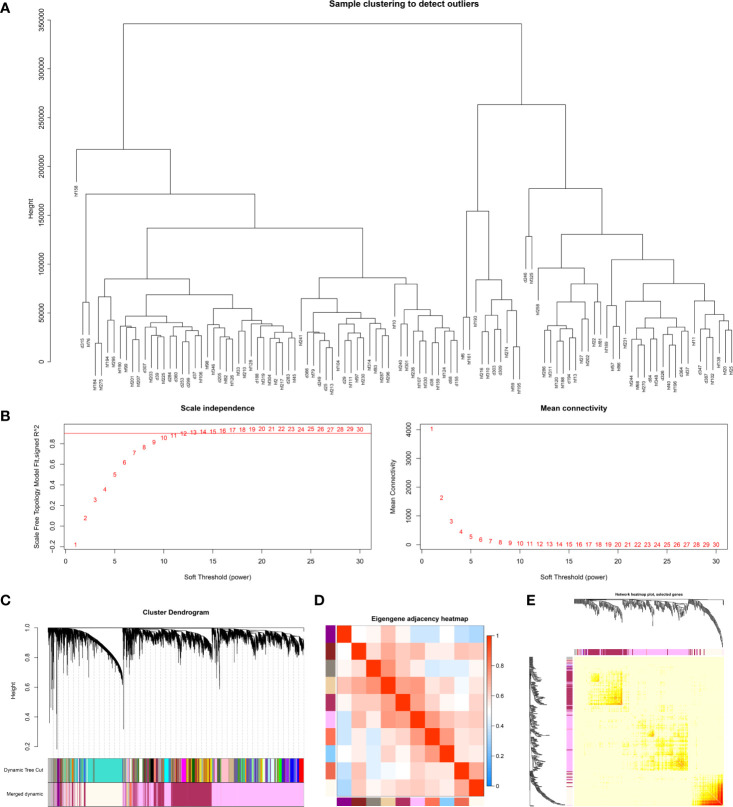
**(A)** Sample clustering to detect outliers. All samples were in the clusters and there was no outlier. **(B)** Analysis of network topology for different soft‐thresholding powers. The soft-thresholding power of 10 was selected for scale-free topology construction. **(C)** Clustering dendrogram of genes with dissimilarity based on the topological overlap, together with assigned module colors. **(D)** Eigengene adjacency heatmap. The color of column and row square represents the adjacency of corresponded modules. In the heatmap, red represents high adjacency (positive correlation), while blue color represents low adjacency (negative correlation). Squares of red color along the diagonal are the meta‐module. **(E)** Network heatmap plot of 1000 randomly-selected genes. Light color represents low overlap and darker color represents higher overlap. The gene dendrogram and module assignment are also shown along the left side and the top.

The input dataset for WGCNA analysis, GSE63042, consisted of the gene expression data from 28 non-surviving sepsis patients and 78 surviving sepsis patients. We Set cut height= 30000 and no abnormalities were detected after sample clustering that warranted their removal (**Figure 1A**). In this study, we chose the power of β=10 for scale-free topology construction due to its scale independence and mean connectivity (**Figure 1B**). Then we set MEDissThres to 0.4 to merge similar modules and obtained a total of eleven modules (M1-11) (**Figure 1C**). Genes that could not be clustered into any modules were put into the M11 module and were identified as non-co-expressed genes.

We analyzed the eigengenes adjacency of the modules in order to determine the proximity of modules (**Figure 1D**). We also performed a network analysis of 1000 randomly selected genes to analyze the interaction relationships among these modules (**Figure 1E**). These two results showed low adjacency between modules, which suggested a large-scale degree of independence in our clustering.

### Identification of Hub Genes and Dataset Validation

**Figure 2:**
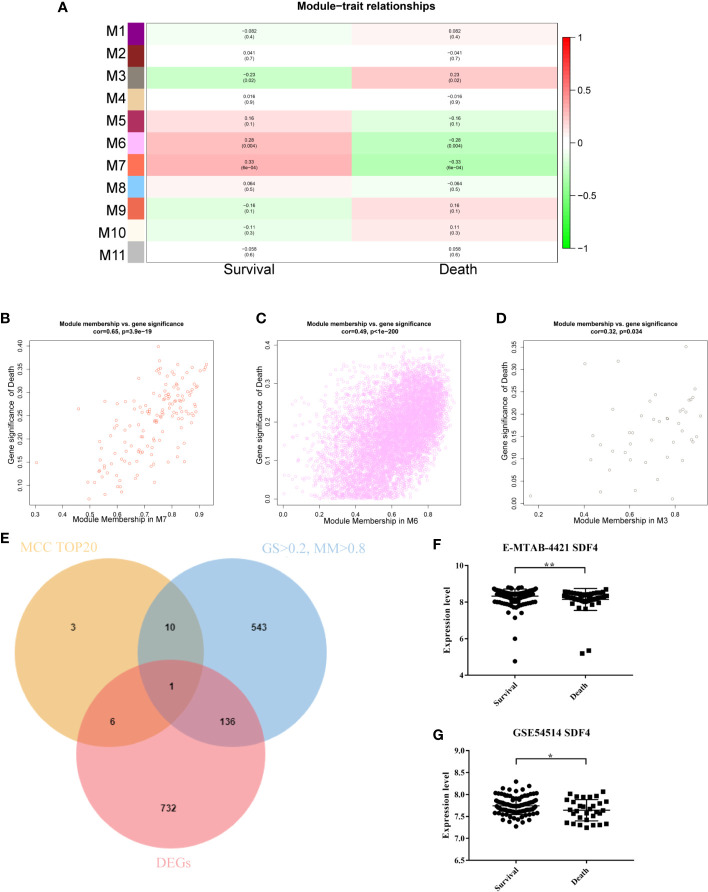
**(A)** Module-trait associations. Each row corresponds to a module eigengene, and each column corresponds to a trait. Each cell contains the corresponding correlation and *P*-value. The table is color-coded by correlation according to the color legend. **(B)** Correlation between module membership and gene significance in the M7 module. **(C)** Correlation between module membership and gene significance in the M6 module. **(D)** Correlation between module membership and gene significance in the M3 module. **(E)** Identification of the hub gene in the intersection of MCC TOP20, DEGs, and GS > 0.2, and MM > 0.8. **(F)** *SDF4* expression in survivors compared to non-survivors in the validation dataset E-MTAB-421. **(G)** *SDF4* expression in survival compared to death in validation dataset GSE54514. **p < 0.01, *p < 0.05.

To identify modules related to the prognosis of sepsis, we performed a heatmap of module-trait relationships (**Figure 2A**). There are two modules (M7, *P* = 6e-04 and M6, *P* = 0.004) significantly associated with positive outcomes when expressed at a higher level, while one module (M3, *P* = 0.016) significantly associated with negative outcomes when expressed at a higher level. Mean gene significance and errors of each module were then calculated and three modules were found to rank in the top three (**Supplementary Figure 1**). Subsequently, we used two parameters, gene significance (GS) and module membership (MM), to assess the relationship between module and trait (**Figures 2B–D**).

A total of 1,238 genes differentially expressed (1,099 annotated) (25). Only 78 DEGs were confirmed in M7 module while 5 DEGs were confirmed in M3. In M6 module, we ultimately identified 875 DEGs, which accounted for 79.6% of the total DEGs. More DEGs in modules indicated more contribution to the trait, therefore we chose the M6 module for subsequent analysis.

In order to identify hub genes in the target module, gene-gene connections with a weight > 0.1 were filtered out and imported to Cytoscape. Then, we calculated the MCC values *via* Cytohubba and constructed a network based on the top 20 genes (**Supplementary Figure 2**). Together with the criteria GS > 0.2, MM > 0.8, and differential expression, *SDF4* was regarded as the hub gene (**Figure 2E**). Finally, *SDF4* was preliminarily validated using other datasets with sepsis prognosis information (GSE54514 and E-MTAB-4421). *SDF4* was also down-regulated in patients who would later die compared to sepsis survivors (**Figures 2F, G**).

### Quantification of SDF4 in Predicting Sepsis Outcome

**Figure 3:**
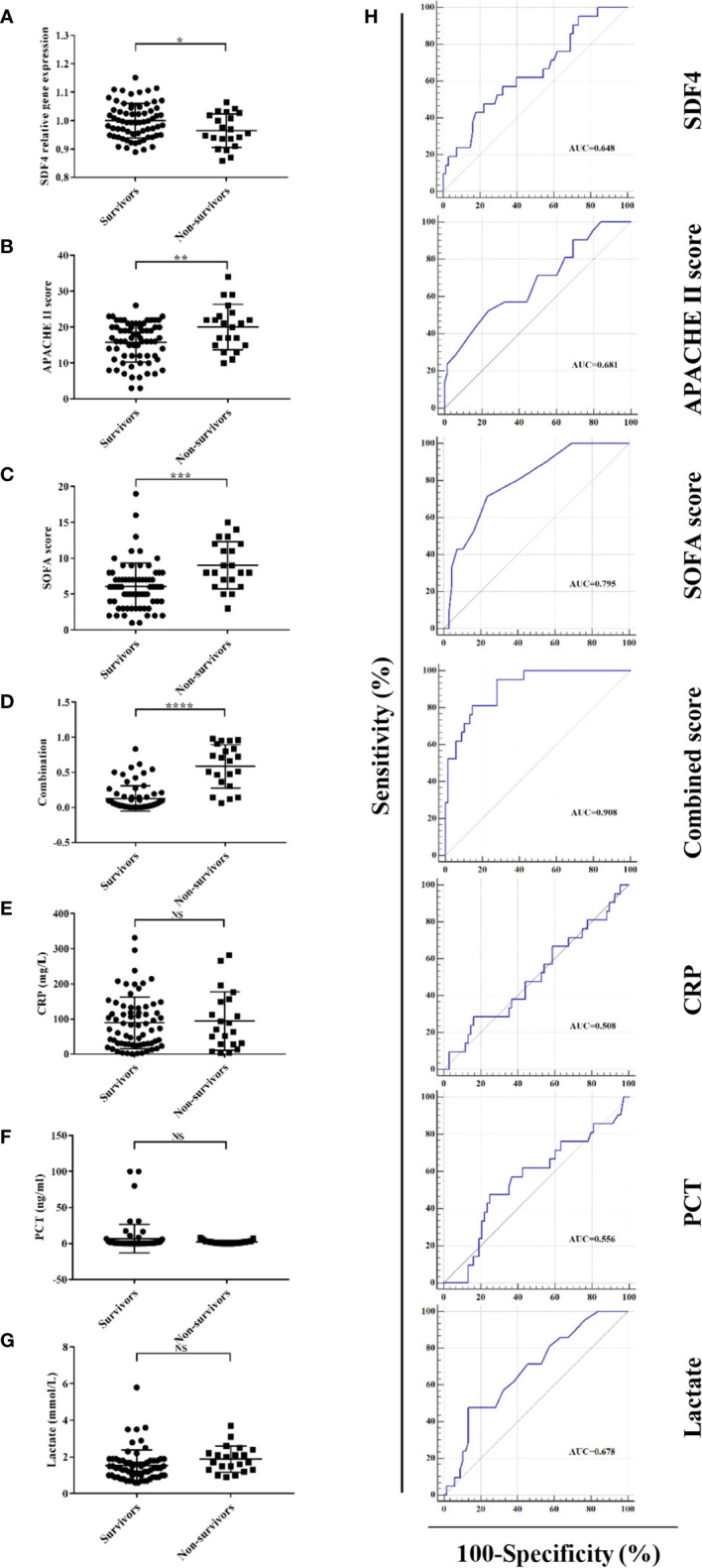
**(A)** *SDF4* expression in survivors (n=68) compared to non-survivors (n=21) within the cohort. **(B)** APACHE II score in survivors compared to non-survivors. **(C)** SOFA score in survivors compared to non-survivors. **(D)** Combined score in survivors compared to non-survivors. **(E)** *CRP* in survivors compared to non-survivors. **(F)** *PCT in* survivors compared to non-survivors. **(G)** Lactate in survivors compared to non-survivors. **(H)** Receiver operating characteristics (ROC) curve of a diagnostic test based on *SDF4* expression, APACHE II score, SOFA score, combined score, *CRP*, *PCT*, and lactate. ****P < 0.0001, ***P < 0.001, **P < 0.01, *P < 0.05. NS, not significant.

A total of 89 patients diagnosed with sepsis after admission to the ICU were in our cohort. **Table 1** shows the baseline demographics and clinical characteristics of survivors and non-survivors. Survivors and non-survivors had average ages of 59.1 and 63.7 respectively. Those patients with cancer (*P* = 0.02) and chronic kidney disease (*P* = 0.01) tended to have worse outcomes within 28 days. Patients who died within 28 days had higher APACHE II scores (20.1 ± 6.2 versus 15.8 ± 5.4, *P* = 0.004) and SOFA scores (9.5 ± 3.1 versus 6.1 ± 3.3, *P* < 0.001) compared to patients who survived when initially admitted to ICU (**Table 1** and **Figures 3B, C**).

**Table 1:** Characteristics of patients included in the study.

Characteristic	Survivors (n=68)	Non-survivors (n=21)	*P* value
Demographics			
Age (years)	59.1 (16.7)	63.7 (14.5)	0.25
Male sex	47 (69.1%)	13 (61.9%)	0.54
Smoking	25 (36.8%)	12 (57.1%)	0.10
Chronic comorbidity			
Hypertension	34 (50.0%)	10 (47.6%)	0.85*
Diabetes	12 (17.6%)	5 (23.8%)	0.53*
Chronic kidney disease	9 (13.2%)	8 (38.1%)	0.01*
Cardiac failure	8 (11.8%)	1 (4.8%)	0.35*
Chronic liver disease	5 (7.4%)	3 (14.3%)	0.33*
Cancer	17 (25.0%)	11 (52.4%)	0.02*
Infection			
Gram-positive bacteria	3 (4.4%)	0 (0%)	0.33*
Gram-negative bacteria	4 (5.9%)	2 (9.5%)	0.56*
Viral	1 (1.5%)	1 (4.8%)	0.37*
Fungus	5 (7.4%)	4 (19.0%)	0.12*
Severity at time of admission to ICU			
APACHE II score	15.8 (5.4)	20.1 (6.3)	**0.004**
SOFA score	6.1 (3.3)	9.5 (3.1)	**<0.001**
Laboratory data			
WBC (×109/L)	12.1 (6.6)	12.6(9.9)	0.80
Neutrophil (%)	85.3 (10.9)	88.9 (6.6)	0.16
Lymphocyte (%)	7.7 (6.5)	7.0 (5.0)	0.65
Monocyte (%)	5.4 (3.5)	4.6 (3.3)	0.39
CRP (mg/L)	89.0 (73.9)	94.2 (83.0)	0.79
PCT (ng/mL)	6.7 (19.8)	2.0 (0.5)	0.29
Lactate (mmol/L)	1.5 (0.8)	1.9 (0.7)	0.09

Data are n (%) or mean (SD). APACHE II, Acute Physiology and Chronic Health Evaluation II; SOFA, Sequential Organ Failure Assessment; CRP, C‐reactive protein; PCT, procalcitonin. Statistical analysis t test unless otherwise specified. *χ² test. Bold denotes statistical difference.

For further validation, we inspected *SDF4* expression level in PBMCs within the cohort and as expected, patients who died had significantly lower *SDF4* expression in comparison with the survivors (0.96 ± 0.01 versus 1 ± 0.01) (**Figure 3A**). To identify the combined signature, we performed a logistic regression analysis. Among all characteristics, cancer, chronic kidney disease, APACHE II score, SOFA score, and *SDF4* expression levels revealed statistical significance (*P* < 0.1) in univariate analysis. With these variables, multivariate analysis was conducted and the result was the following: Combined score = -23.845 * (*SDF4* relative expression level) + 0.123 * (APACHE II score) + 0.323*(SOFA score) + 3.183 * (cancer status: 0 for without, 1 for with) + 1.988 * (chronic kidney disease: 0 for without, 1 for with) + 15.789 (**Table 2** and **Figure 3D**). ROC analysis was constructed to examine the performance of indicators as predictors of outcome, and the AUC for each indicator was calculated, respectively (**Figure 3H**). *PCT* (procalcitonin), *CRP* (C-reactive protein), and lactate were previous reported as biomarkers for prognosis (32–34). Thus, these three variables were also analyzed in the study (**Figures 3E–H**). The AUC of the expression level of *SDF4* (AUC = 0.648) is superior to *CRP* (AYC = 0.508) and *PCT* (AUC = 0.556), similar to APACHE II score (AUC = 0.681) and lactate (AUC = 0.678), and slightly inferior to the SOFA score (AUC = 0.795). The combined score (AUC = 0.908) had the highest power to discriminate between the two groups, which improved the predictive value of model only with clinical parameters (APACHE II score+ SOFA score+ CKD + cancer, AUC= 0.828).

**Table 2:** Results of multivariate logistic regression modeling.

Characteristic	n = 89		
	HR	95% CI	*P* value
SDF4 expression level	0.165	0.054-0.508	0.002
APACHE II score	1.131	0.982-1.302	0.088
SOFA score	1.381	1.126-1.695	0.002
Cancer	24.062	3.707-156.183	0.001
Chronic kidney disease	7.289	1.404-37.857	0.018

HR, hazard ratio; CI, confidence interval.

**Figure 4:**
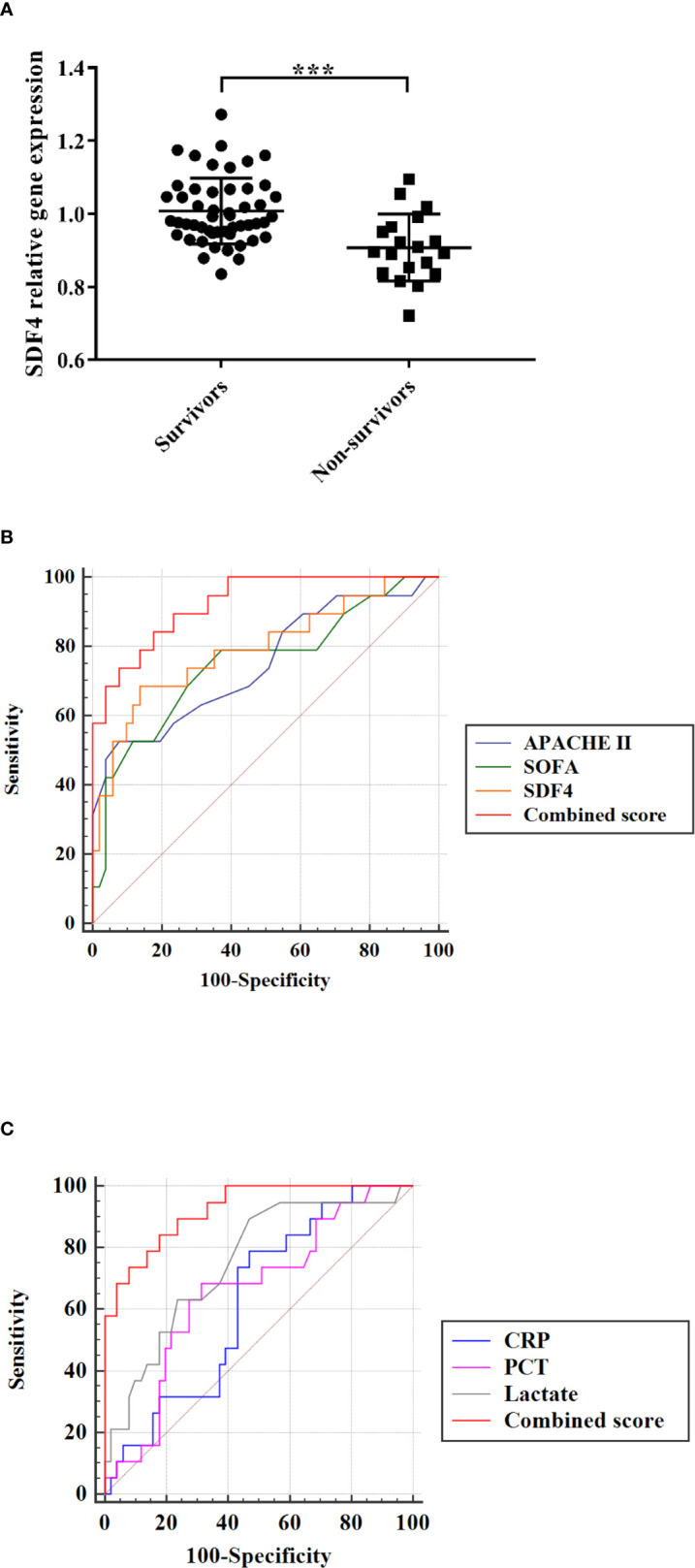
**(A)** *SDF4* expression in survival (n=51) compared with death (n=19) in validation cohort. **(B)** Receiver operating characteristics (ROC) curve of a diagnostic test based on SDF4 (AUC=0.794), APACHE II score (AUC=0.750), SOFA score (AUC=0.751) and combined score (AUC=0.925). **(C)** Receiver operating characteristics (ROC) curve of a diagnostic test based on *CRP* (AUC=0.624), *PCT* (AUC=0.659), lactate (AUC=0.752) and combined score. ***p < 0.001.

To evaluate the performance of the model, 70 patients diagnosed with sepsis after admission to the ICU were involved and RNA from their PBMCs were extracted. Patients who died (n=19) had significantly lower SDF4 expression in comparison with the survivors (n=51) (**Figure 4A**). The AUC of combined score is superior to APACHE II score, SOFA score (**Figure 4B**) and laboratory parameter (**Figure 4C**).

### Endoplasmic Reticulum Stress May Be the Downstream Effect of SDF4

**Figure 5:**
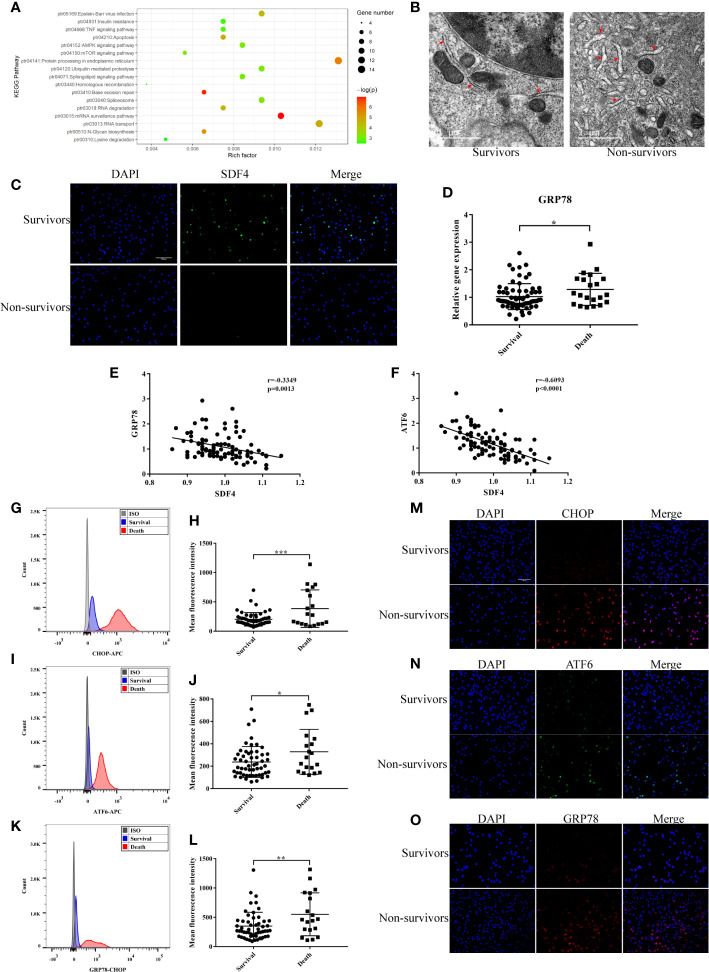
**(A)** Kyoto Encyclopedia of Genes and Genomes (KEGG) enrichment analysis of DEGs (p < 0.05). **(B)** Representative TEM picture in survival and death. **(C)** Representative photomicrographs showing *SDF4* (green), DAPI (blue) and their merged images (original magnification ×400). Scale bar, 100um. **(D)** *GRP78* expression in survival (n=68) compared with death (n=21) in cohort. **(E)** Pearson correlation analysis between the expression level of *GRP78* and *SDF4 in* cohort. **(F)** Pearson correlation analysis between the expression level of *ATF6* and *SDF4* in cohort. **(G)** Representative histograms of *CHOP* expression among survival and death in flow cytometry. **(H)** Mean fluorescence intensity of *CHOP* in survival (n=54) and death (n=19). **(I)** Representative histograms of *ATF6* expression among survival and death in flow cytometry. **(J)** Mean fluorescence intensity of *ATF6* in survival and death. **(K)** Representative histograms of *GRP78* expression among survival and death in flow cytometry. **(L)** Mean fluorescence intensity of *GRP78* in survival and death. **(M)** Representative photomicrographs showing *CHOP* (red), DAPI (blue) and their merged images (original magnification ×400). Scale bar, 100um. **(N)** Representative photomicrographs showing *ATF6* (green), DAPI (blue) and their merged images (original magnification ×400). **(O)** Representative photomicrographs showing *GRP78* (red), DAPI (blue) and their merged images (original magnification ×400). ***p < 0.001, **p < 0.01, *p < 0.05.

KEGG pathway enrichment was performed for M6 module for functional annotation. Protein processing in the endoplasmic reticulum (ER) was the pathway with the highest enrichment factor (**Figure 5A** and **Supplementary Table 2**).

Alternative splicing of *SDF4* encoded protein, Cab45, produces three different isoforms: Cab45C, Cab45G, and Cab45S (35). Cab45C is reported to be a cytosolic splice variant and participates in Ca^2+^-induced amylase secretion (36). Cab45G is localized to the Golgi lumen, required for Ca^2+^-dependent cargo sorting at the trans-Golgi network, and can regulate impairment elicited by ethanol or UV (37–39). Cab45S, is related to ER Ca^2+^ signaling (40) and ER stress response genes (41, 42). Therefore, we speculated that the underlying mechanism of *SDF4* is associated with ER function. Firstly, PBMCs were observed with transmission electron microscopy (TEM). Those patients with negative outcomes showed more severe swelling of the ER and ribosome degranulation (**Figure 5B**). After the expression level of SDF4 was verified (**Figure 5C**), we next determined the expression levels of *GRP78*, *CHOP*, and *ATF6* in our cohort, which are key genes in signaling cascades to reduce the accumulation of unfolded proteins. We found that patients who died within 28 days tended to express *GRP78* more highly (1.29 ± 0.13 versus 1.03 ± 0.06, **Figure 5D**). Moreover, we evaluated the correlation between *SFD4* expression and *GRP78* expression in PBMCs and found that *GRP78* expression was negatively correlated with *SDF4* (**Figure 5E**). Similar results were observed for the correlation between *SDF4* and *ATF6* expression (**Figure 5F**). In addition, our data showed that *GRP78*, *CHOP*, and *ATF6* were over-expressed in patients with severe sepsis that led to death *via* calculating the mean fluorescence intensity in flow cytometry (**Figures 5G–L**), which was further verified by immunofluorescence (**Figures 5M–O**) in another cohort. Taken together, our data indicate that ER stress may be up-regulated as a result of down-regulation of *SDF4*, leading to worsened sepsis outcomes.

### Sdf4 Overexpression Inhibited Endoplasmic Reticulum Stress in CLP Mice Lung

**Figure 6:**
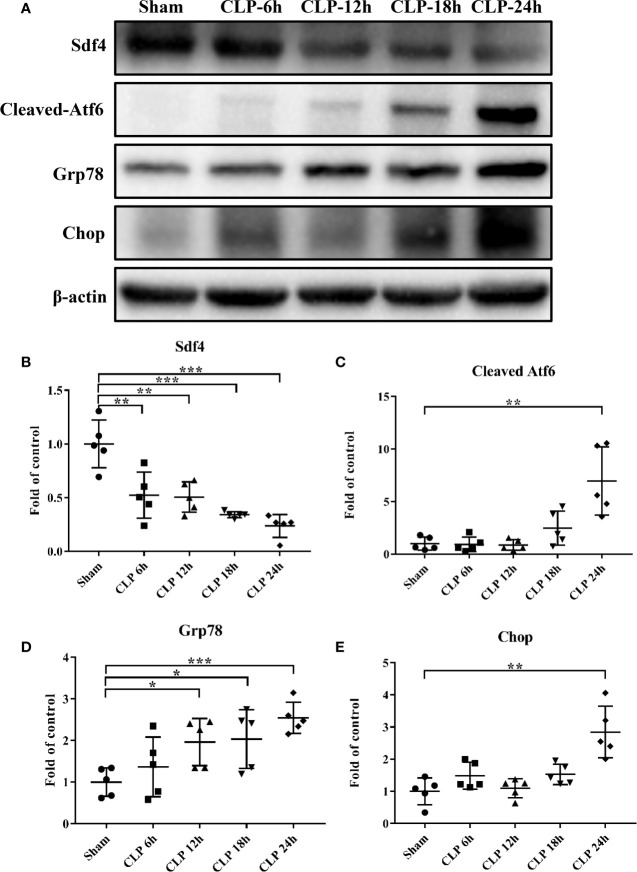
**(A)**. Representative western blot results from sham-operated and CLP mice lung for levels of *cleaved- Atf6*, *Grp78*, *Chop*, and β-actin. **(B–E)** The *cleaved- Atf6*, *Grp78* and *Chop* bands were quantified by densitometry and normalized to the density of β-actin. n=5. Data were shown in Mean ± SD. ***p < 0.001, **p < 0.01, *p < 0.05.

Considering the decreased expression of *SDF4* in sepsis patients, we investigated whether *Sdf4* was down-regulated in CLP-induced sepsis in mice. Acute lung damage is one of the main causes of death among sepsis patients, thus we selected lung as the target organ. We first detected the level of *Sdf4* expression and ER stress of lungs 6 h, 12 h, 18 h and 24 h following our CLP models. We found that Sdf4 gradually decreased along with an increasing of *cleaved-Atf6*, *Grp78* and *Chop* as the disease progressing (**Figure 6**). Therefore, 24 h after CLP was selected as the observation point in the following study.

**Figure 7:**
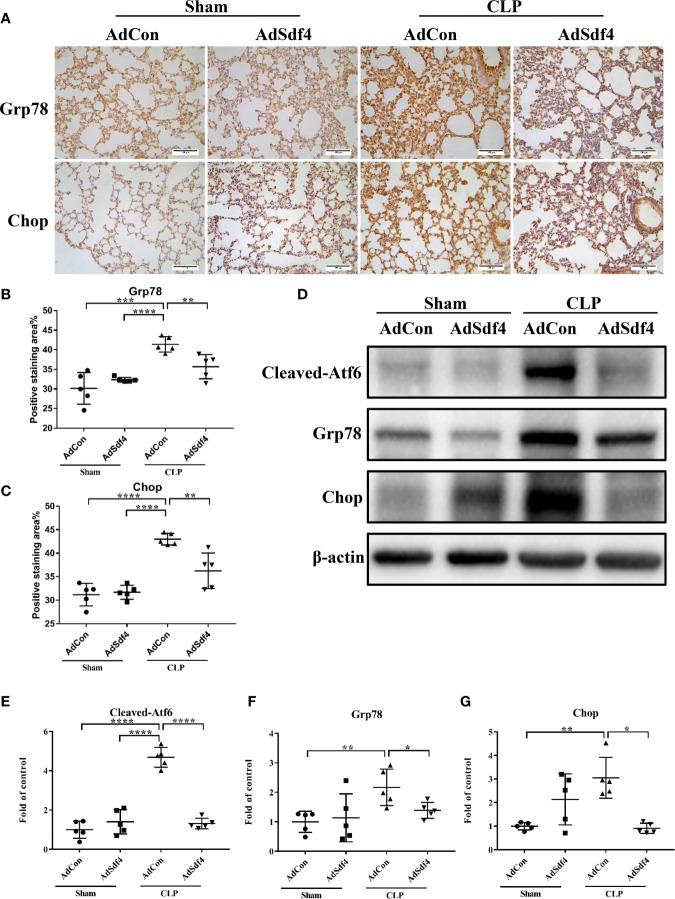
**(A)** Representative photomicrographs of *Grp78* and *Chop* from sham-operated and CLP mice lung treated with AdCon or AdSdf4 (original magnification ×400). Scale bar, 100um. **(B, C)** Positive staining area of *Grp78* and *Chop* from four groups. **(D)** Representative western blot results from four groups for levels of *cleaved- Atf6*, *Grp78*, *Chop*, and β-actin. **(E–G)** The *cleaved- Atf6*, *Grp78* and *Chop* bands were quantified by densitometry and normalized to the density of β-actin. n=5. Data were shown in Mean ± SD. ****p < 0.001, ***p < 0.001, **p < 0.01, *p < 0.05.

To determine the role of *Sdf4* in sepsis-induced lung injury, we overexpressed *Sdf4* using adenovirus vector. Each mouse was intratracheally treated with 10^10^ plaque-forming units (PFU) of recombinant *Sdf4* adenovirus (AdSdf4), EGFP adenovirus (AdCon) or PBS and allowed to recover for 72 hours. We first confirmed adenoviral gene delivery and transfection. EGFP signals were detected in both AdSdf4 and AdCon group (**Supplementary Figure 3A**). *Sdf4* overexpression is verified by immunofluorescence (**Supplementary Figure 3B**) and western blot (**Supplementary Figures 3C, D**). Then, AdSdf4 and AdCon mice were subjected to CLP-induced sepsis and were sacrificed 24h after CLP. IHC staining against *Grp78* and *Chop* showed ER stress was down-regulated in AdSdf4 mice compared with AdCon mice (**Figures 7A–C**). Western blot presented a similar change (**Figures 7D–G**).

Considered together, these results indicated that the *Sdf4* is a negative upstream regulator of ER stress in sepsis-induced lung injury, whereas overexpression of *Sdf4* attenuated excessive ER stress.

## Discussion

Sepsis is a life-threatening complication caused by infections, leading to severe host response dysregulation and organ dysfunction (1). A recent study has revealed that sepsis is a complex and dynamic syndrome with great heterogeneity (43). Therefore, co-expression network analysis was employed in the current study to mine the hub gene and explore the underlying mechanisms of sepsis responses in the current study. Expression data of 28 sepsis non-survivors and 78 sepsis survivors were used for WGCNA analysis. Through WGCNA, we divided all genes into 11 separate modules and found that the M6 module was indicative of sepsis outcome. *SDF4* was then screened out satisfying three criteria: 1) DEGs; 2) GS > 0.2, MM > 0.8; 3) the 20 highest MCC value, followed by validation in both two datasets and clinical cohorts. Evidence from KEGG and a literature review further demonstrated the potential relationship between *SDF4* expression and ER stress.

For sepsis patients, screening for risk factors and prognosis could have a role in triaging direct resources appropriately to the most vulnerable patients (44). Meaningful definitions of sepsis severity are supposed to capture a subset of patients where severe physiological derangements led to a substantially great mortality risk (45–47). In addition, more than 100 biomarkers have been evaluated for sepsis prognosis in the last few decades (6, 7). Regardless of this, no gold standard for prognosis yet exists and clinicians still rely on a number of traditional biomarkers to discriminate between patients with positive and negative outcomes. Reportedly, *PCT*, *CRP*, lactate levels, and SOFA scores have a moderate predictive value in sepsis prognosis (33, 48, 49), while prognosis accuracy depends much on multiple assessments and measurements throughout disease progression. Our findings showed that *SDF4* expression level was significantly lower in non-survivors compared to survivors and that its prediction value is superior to that of *CRP* and *PCT*, equal to lactate and the APACHE II score, and inferior to the SOFA score. As a combination of several sepsis biomarkers might be more effective (6), we applied a multivariable logistic regression approach and the combined score turned out to be a satisfactory predictor with an AUC of 0.908. Incorporation of *SDF4* can improve clinical parameters predictive value for the prognosis of sepsis, showing a potential for future applications.

*SDF4*, mapping to 1p36.33, is a member of the CREC family (50). Previous DNA methylation analysis revealed that the promoter region of *SDF4* was hypomethylated in porcine mammary epithelial cells faced with *Escherichia coli* challenge (51), suggesting the potential effect of *SDF4* in sepsis. Alternative splicing of its encoded protein, Cab45, produces three different isoforms: Cab45C, Cab45G, and Cab45S (35). Cab45C is reported to be a cytosolic splice variant and participates in Ca^2+^-induced amylase secretion (36). Cab45G is localized to the Golgi lumen, required for Ca^2+^-dependent cargo sorting at the trans-Golgi network, and can regulate impairment elicited by ethanol or UV (37–39). Cab45S, a secreted variant of Cab45, can inhibit ER stress and apoptosis *via* GRP78/Bip and can promote cell proliferation through inhibition of Ca^2+^ signaling in tumors (40, 42). The downregulation of apoptosis was also observed in neurons when *SDF4* was overexpressed (41). We observed that *SDF4* was a protective factor for sepsis patients in both the validation datasets and cohort. KEGG pathway analysis also revealed protein processes in the ER as the most enriched pathway in the M6 module, so we shifted our focus to ER dysfunction.

The ER is a vital intracellular organelle in eukaryotic cells, which is constructed of a continuous membrane system of tubules and sheets (52). The ER plays a major role in protein translocation, folding, post-translational modifications, and transportation to the Golgi body, commonly considered as a protein folding factory (53). The ER also serves a role in calcium storage and lipid metabolism (54). Accumulated evidence supports that the ER is altered in septic pathology and ER stress is involved in the progression of sepsis, which is marked by the accumulation of misfolded or unfolded proteins (55). ER stress not only contributes to abnormal lymphocyte apoptosis (56), but also impairs beneficial autophagy activity in septic mice (57). In addition, it was reported that ER stress can modulate the NF-κB/IκB and HIF-1α signaling pathways in LPS-induced lung inflammation (58). Nevertheless, there is little research focusing on the relation between ER stress and sepsis prognosis. In the current study, *ATF6*, *GRP78*, and *CHOP* were significantly overexpressed in non-survivors compared to survivors, which was confirmed in both flow cytometry and immunofluorescence. And to further verify our hypothesis, we established the mice CLP model and found that ER stress gradually increased within 24 hours after surgery, accompanied by the decreasing of *Sdf4*. And overexpression of Sdf4 by intratracheally injecting recombinant adenovirus attenuated such activation. In mild or temporary ER stress, this signaling cascade can restore the cell to homeostasis. Conversely, if stress is long-term or excessive and cellular function is compromised, apoptosis is initiated involving the transcription factor C/EPB homologous protein (CHOP) (59).

In conclusion, using WGCNA analysis, our study identified *SDF4* as a biomarker of sepsis prognosis, and a prediction model was established based on *SDF4* expression levels and clinical characteristics. Then, the mechanism was speculated and preliminarily verified in sepsis patients and CLP mice. Upon inflammatory overload in severe sepsis, the *SDF4* expression level was decreased, leading to an ER stress signaling cascade. ER stress beyond the regulation level of cells results in compromised cellular function, which gives rise to a worsened outcome. Nevertheless, the study also has some limitations. Firstly, the performance of the combined score for sepsis outcome prediction requires an evaluation in a larger multicenter and prospective study to compare with existing biomarkers. Secondly, we only followed up for 28 days but did not confirm the impact of *SDF4* expression and ER stress levels on the long-term mortality of septic patients. Finally, studies elucidating dynamic changes in SDF4 and ER stress with disease process or therapeutic intervention and the molecular mechanism of *SDF4* and ER stress in sepsis *in vivo* or *in vitro* might be of great significance in the future.

## Data Availability Statement

Publicly available datasets were analyzed in this study. This data can be found here: GSE63042, GSE54514 and E-MTAB-4421.

## Ethics Statement

The studies involving human participants were reviewed and approved by the Research Ethics Committee of the First Affiliated Hospital, College of Medicine, Zhejiang University. The patients/participants provided their written informed consent to participate in this study. The animal study was reviewed and approved by Research Ethics Committee of the First Affiliated Hospital, College of Medicine, Zhejiang University.

## Author Contributions 

JC, HJ, TZ, and QS designed the study. TZ and HC analyzed bioinformatics. QS, XP, and SC recruited sepsis patients cohort and collected clinical characteristics. TZ, CW, SF, and LS carried out experiments. QS and YW analyzed data. All authors contributed to the article and approved the submitted version.

## Funding

This work was supported by the key research project of precision medicine in National Key Research and Development Plan (2017YFC0907603), and Natural Science Foundation of China (81900611), LQ19H050008, 2018M642464.

## Conflict of Interest

The authors declare that the research was conducted in the absence of any commercial or financial relationships that could be construed as a potential conflict of interest.

## Supplementary Material

Supplementary Figure 1 Distribution of average gene expression significance and errors in the modules associated with sepsis outcome.

Supplementary Figure 2 The network contains the top 20 genes in the MCC of the M6 module. The gradation of the color represents the MCC score and the width represents the weight of each two nodes.

Supplementary Figure 3
**(A)** Representative photomicrographs showing EGFP from mice treated with PBS, AdCon and AdSdf4. **(B)** Representative photomicrographs showing *Sdf4* (red), DAPI (blue) and their merged images from mice treated with PBS, AdCon and AdSdf4. **(C)** Representative western blot results from mice treated with PBS, AdCon and AdSdf4 for levels of *Sdf4* and β-actin. **(D)** The *Sdf4* bands were quantified by densitometry and normalized to the density of β-actin. n=5. Data were shown in Mean ± SD. **p < 0.01.
