# Supplementary figures and images for: SDF4 Is a Prognostic Factor for 28-Days Mortality in Patients With Sepsis via Negatively Regulating ER Stress
Source: Front Immunol. 2021 Jul 13;12:659193. doi: 10.3389/fimmu.2021.659193 (PMC8313857; doi:10.3389/fimmu.2021.659193)

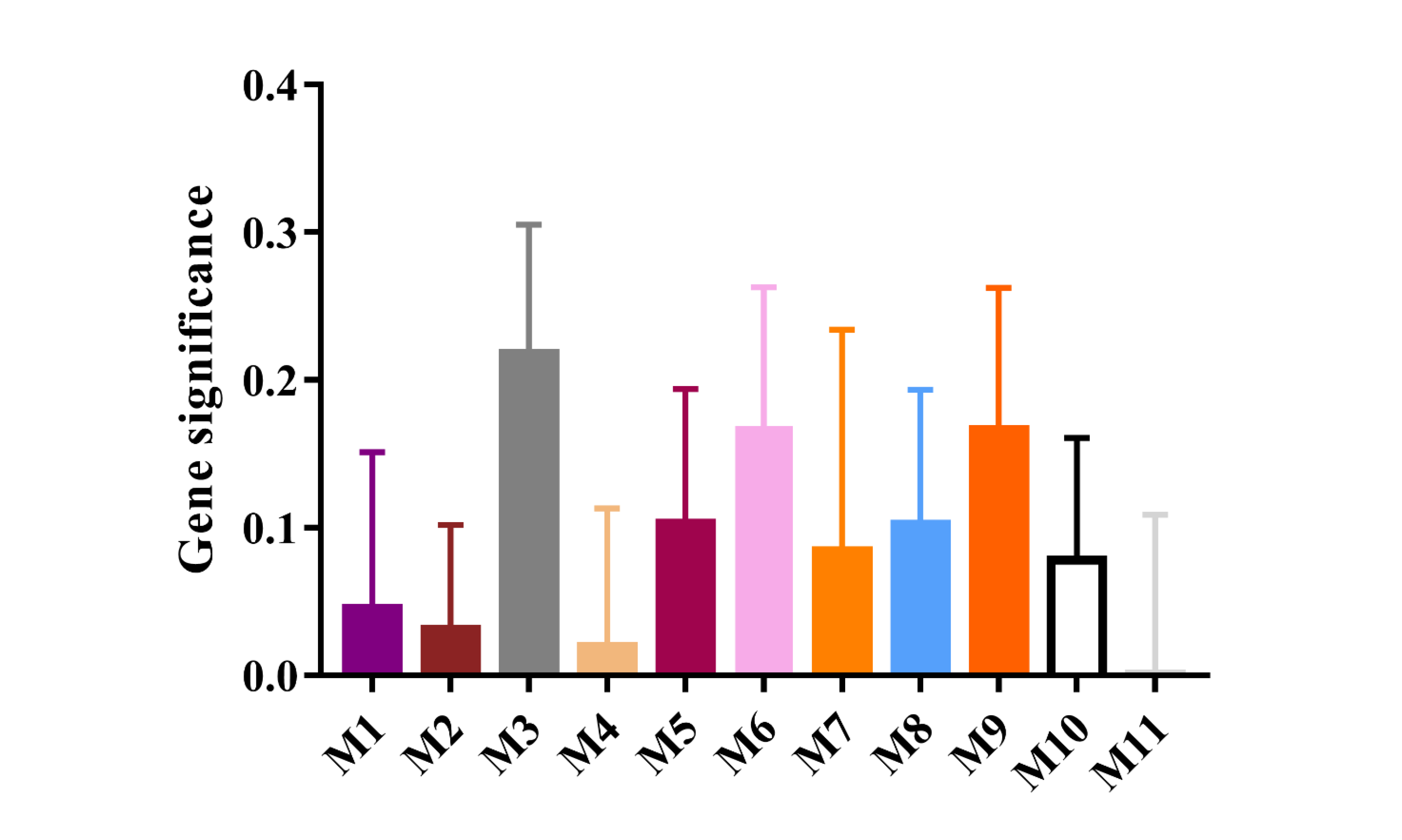

Supplement: Supplementary Figure 1 — Distribution of average gene expression significance and errors in the modules associated with sepsis outcome. [file Image_1.tif]

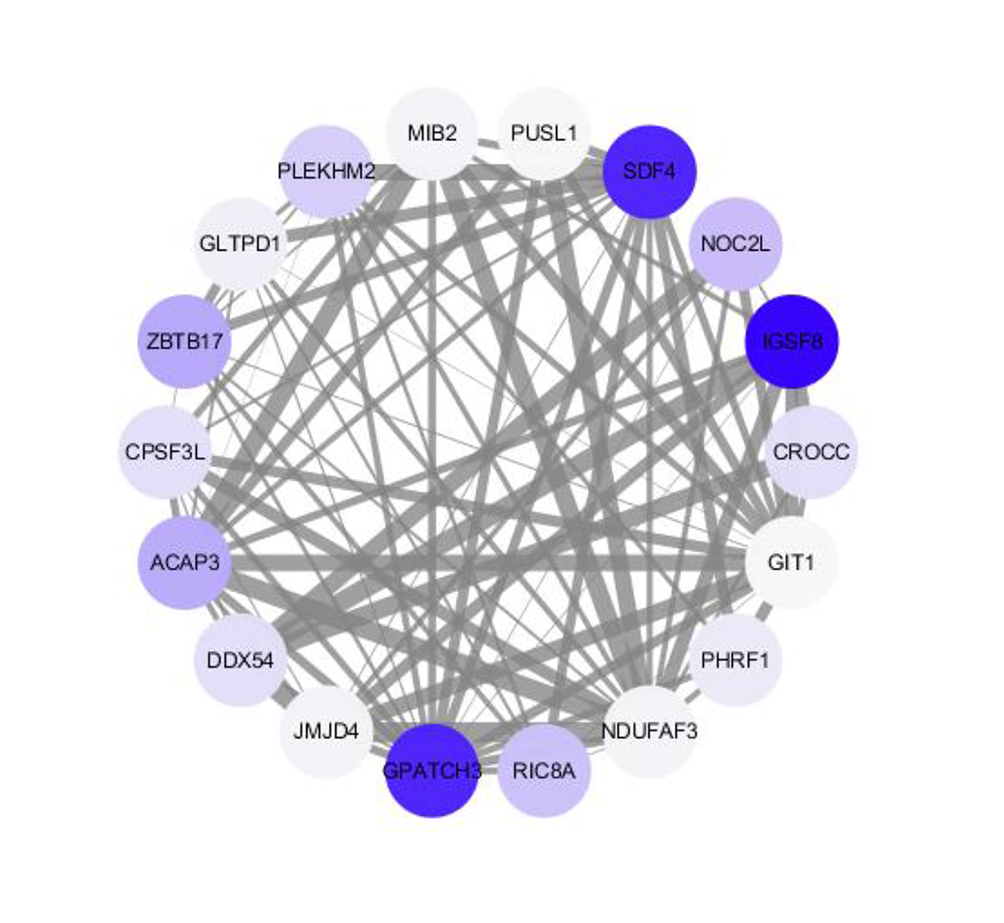

Supplement: Supplementary Figure 2 — The network contains the top 20 genes in the MCC of the M6 module. The gradation of the color represents the MCC score and the width represents the weight of each two nodes. [file Image_2.tif]

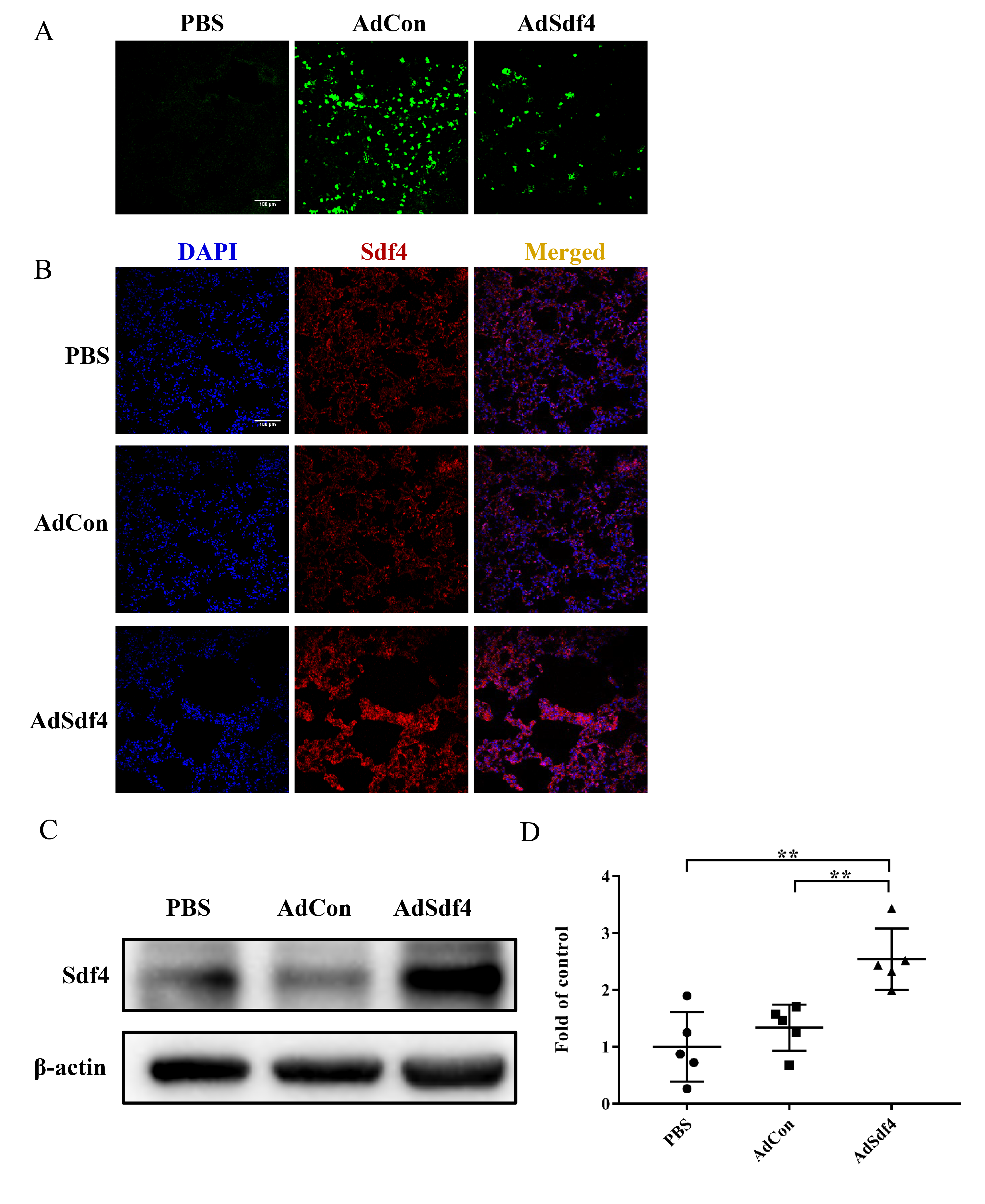

Supplement: Supplementary Figure 3 — (A) Representative photomicrographs showing EGFP from mice treated with PBS, AdCon and AdSdf4. (B) Representative photomicrographs showing Sdf4 (red), DAPI (blue) and their merged images from mice treated with PBS, AdCon and AdSdf4. (C) Representative western blot results from mice treated with PBS, AdCon and AdSdf4 for levels of Sdf4 and β-actin. (D) The Sdf4 bands were quantified by densitometry and normalized to the density of β-actin. n=5. Data were shown in Mean ± SD. **p < 0.01. [file Image_3.tif]
